# Mannose phosphotransferase system subunit IID of *Streptococcus mutans* elicits maturation and activation of dendritic cells

**DOI:** 10.71150/jm.2505014

**Published:** 2025-10-31

**Authors:** Sungho Jeong, Chaeyeon Park, Dongwook Lee, Hyun Jung Ji, Ho Seong Seo, Cheol-Heui Yun, Jintaek Im, Seung Hyun Han

**Affiliations:** 1 Department of Oral Microbiology and Immunology, and Dental Research Institute, School of Dentistry, Seoul National University, Seoul 08826, Republic of Korea; 2 Research Division for Biotechnology, Korea Atomic Energy Research Institute, Jeongeup 56212, Republic of Korea; 3 Department of Agricultural Biotechnology, and Research Institute of Agriculture and Life Sciences, Seoul National University, Seoul 08826, Republic of Korea; 4 Institutes of Green Bio Science and Technology, Seoul National University, Pyeongchang 25354, Republic of Korea

**Keywords:** *Streptococcus mutans*, mannose phosphotransferase system subunit IID, dendritic cells, bacterial adherence and internalization, maturation and activation of dendritic cells

## Abstract

*Streptococcus mutans* is a Gram-positive pathogen that causes dental caries and subsequent pulpal infection leading to pulpitis. Although dendritic cells (DCs) are known to be involved in disease progression and immune responses during *S. mutans* infection, little is known about which component of *S. mutans* is responsible for the DC responses. Although the mannose phosphotransferase system (Man-PTS) is the primary sugar transporter of *S. mutans*, it is also a potential virulence factor. Since Man-PTS subunit IID (ManIID) embedded on the bacterial membrane is indispensable for Man-PTS function, we investigated its role in the maturation and activation of DCs stimulated with a ManIID-deficient strain (Δ*pts*) of *S. mutans* and recombinant ManIID (rManIID) protein. When mouse bone marrow-derived DCs were treated with heat-killed *S. mutans* wild-type (WT) or Δ*pts*, bacterial adherence and internalization of Δ*pts* were lower than those of WT. Moreover, the heat-killed *S. mutans* Δ*pts* strain was inferior to the wild-type in inducing expression of phenotypic maturation markers, such as CD80, CD86, MHC-I, and MHC-II, and proinflammatory cytokine, IL-6. In line with the trends in marker expression, the endocytic capacity of DCs treated with the Δ*pts* strain was comparable to that of untreated DCs whereas DCs treated with the WT strain dose-dependently lost their endocytic capacity. Furthermore, rManIID dose-dependently promoted both phenotypic maturation marker expression and IL-6 production by DCs. Collectively, these results demonstrate that ManIID plays a crucial role in the adhesion and internalization of *S. mutans* into DCs and is one of the major immune-stimulating agents responsible for maturation and activation of DCs during *S. mutans* infection.

## Introduction

Dendritic cells (DCs) are antigen-presenting cells that recognize, capture, and present antigens to naïve T cells, leading to activation of antigen-specific adaptive immunity (Yin et al., 2021). In general, immature DCs (iDCs) are highly endocytic, enabling them to maximize their capacity to capture and uptake various antigens (Reis e Sousa et al., 1999). These processes are mainly mediated by endocytic receptors, including cluster of differentiation (CD) 205 and CD206 (Zhang et al., 2006). Moreover, since CD206 is a mannose receptor, its high expression level on iDCs facilitates the capture and internalization of mannosylated antigens (Wollenberg et al., 2002). Under bacterial infectious conditions, iDCs initially sense and respond to various microbe-associated molecular patterns (MAMPs) of invading pathogens and mature, resulting in an attenuated ability to capture antigen (Dudek et al., 2013). At maturation, DCs upregulate the expression of costimulatory molecules, including CD80, CD86, and major histocompatibility complex (MHC)-II (Kim et al., 2007; Lapteva et al., 2007), and produce various cytokines essential for triggering the adaptive immune response (Yin et al., 2021).

*Streptococcus mutans* is one of the most frequently found streptococcal species in the oral cavity of humans, accounting for approximately 39% of all oral streptococci (Ikeda and Sandham, 1971). *S. mutans* plays a prominent role in the development of dental caries by creating excessive acid (Forssten et al., 2010; Ikeda and Sandham, 1971; Lemos et al., 2019; Matsumoto-Nakano, 2018). Furthermore, entry of *S. mutans* into dentin following disruption of the tooth enamel by caries exacerbates pulpitis by strengthening dental pulp inﬂammation (Hahn and Liewehr, 2007). In fact, *S. mutans* induces the production of inflammatory cytokines, such as TNF-α, IL-1, and IL-6 by dental pulp cells (Akamp et al., 2024; Maisonneuve et al., 2020). Additionally, *S. mutans* has been implicated in systemic diseases, such as infective endocarditis after gaining access to the bloodstream (Ikeda and Sandham, 1971; Kojima et al., 2012; Nobbs, 2017). Several studies have demonstrated that DCs are important in disease progression and immune responses in *S. mutans* infection through recognition of pathogens and production of various cytokines that bridge host innate and adaptive immunity (Butcher et al., 2011; Hahn et al., 2005).

Streptococci commonly possess a sugar phosphotransferase system (PTS) on their surfaces, which phosphorylates and transports more than 20 carbohydrate substrates into the bacterial cell (Sato et al., 2015). PTS consists of Enzyme I, histidine-containing phosphocarrier protein, and Enzyme Ⅱ containing multiple subunits, such as cytoplasmic ⅡA and ⅡB and/or membrane-embedded ⅡC and ⅡD (Oiki et al., 2019). Accumulated studies point out that PTS may play a prominent role as a potential virulence factor. For example, the virulence of *Salmonella* was shown to be regulated by a PTS mutant with down-regulation of genes related to quorum sensing, flagella, and pathogenicity islands (Lim et al., 2019). Furthermore, PTS was shown to guide the assembly of the capsular polysaccharide of *Klebsiella pneumoniae*, conferring phagocytic resistance against macrophages (Panjaitan et al., 2021). Therefore, PTS is of clinical importance due to its abundance and ability to confer virulence.

Among PTS families, mannose-PTS (Man-PTS) is considered as the primary sugar transporter of *S. mutans* (Ajdić and Pham, 2007). Man-PTS participates in sugar uptake and affects various other biological processes, including acid production, biofilm formation, and glucosyltransferase gene expression (Abranches et al., 2006; Zeng et al., 2017). Moreover, Man-PTS can be structurally distinguished from other PTS families by the presence of subunit ⅡD (ManIID), which is located at the interface of the bacterial membrane (Oiki et al., 2019). According to a previous study, the ManIID is indispensable for the aforementioned biological functions of Man-PTS and critical for the transportation and phosphorylation of sugars, bacteriocin sensitivity as a receptor, and adaptation to the external environment by modulating the metabolic state of the pathogen (Jeckelmann and Erni, 2020). A recent study indicated that mannose metabolism, PTS, and glycolysis pathways were upregulated in patients with periodontitis and type 2 diabetes, suggesting that Man-PTS and its membrane component ManIID is involved in disease progression (Lu et al., 2022). Nevertheless, it has yet to be clarified how this molecule shapes immune responses in the context of DCs. Therefore, in this study, we examined whether ManIID affects the phenotypic and functional activation of DCs in response to wild-type *S. mutans*, a ManIID-deficient strain (Δ*pts*) of *S. mutans*, or recombinant ManIID (rManIID) protein.

## Materials and Methods

### Reagents and chemicals

Recombinant murine granulocyte macrophage colony-stimulating factor (GM-CSF) was obtained from PeproTech. Fetal bovine serum (FBS) was purchased from GIBCO. Ultrapure lipopolysaccharide (LPS) from *Escherichia coli* was purchased from InvivoGen. RPMI-1640 was purchased from Welgene. Isopropyl β-D-1-thiogalactopyranoside (IPTG) was purchased from Biosesang. Tryptic soy broth (TSB) and Todd-Hewitt broth were purchased from BD Biosciences. Luria broth (LB) was obtained from LPS Solution. Red blood cell (RBC) lysis buffer, carboxyfluorescein succinimidyl ester (CFSE), fluorescein isothiocyanate (FITC)-dextran, and mannan from *Saccharomyces cerevisiae* were purchased from Sigma-Aldrich. Phycoerythrin (PE) anti-mouse CD80, FITC anti-mouse CD86, allophycocyanin (APC) anti-mouse MHC-I, PE/Cyanine7 anti-mouse MHC-II, and peridinin-chlorophyll-protein (PerCP) anti-mouse CD11c antibodies were all purchased from BioLegend.

### Generation of a Δ*pts* *S. mutans* strain

The *S. mutans* ATCC 25175 wild-type (WT) strain was obtained from the American Type Culture Collection. *S. mutans* ATCC 25175-derived ManIID-deficient strain (Δ*pts*) was generated by replacing the ManIID gene with the chloramphenicol acetyltransferase (cat) gene through using SnapGene software (Biotech). Briefly, to amplify upstream and downstream flanking regions of the ManIID gene of *S. mutans* ATCC 25175 (D820_08715), gDNA from *S. mutans* ATCC 25175 was amplified using a nPfu DNA polymerase (Enzynomics) with primers containing KpnI or XhoI site (Forward: 5’- TCGGTACCCCTAGGAGACATTAAGACAGG-3’; Reverse: 5’-TCCTCGAGCTATTTAAATATCCTCCTCA-3’), or primers containing BamHI or NotI site (Forward: 5’-GAGGATCCACACAAGGACAAGCCATT-3’; Reverse: 5’-TTGCGGCCGCTTATTTAGAATTGGCAAGTCGC-3’) under the following reaction condition: amplification by 30 cycles at 95°C for 30 s, 56°C for 30 s, and 72°C for 60 s. The PCR products were digested with restriction enzymes (KpnI and XhoI for upstream, and BamHI and NotI for downstream flanking region PCR product) together with pC326 plasmid for 30 min at 37°C. The digested PCR products were cloned into the multiple cloning sites of pC326 plasmid using T4 DNA ligase (New England Biolabs) for 1 h at room temperature. For generating the Δ*pts* strain, the pC326 plasmids containing the flanking regions of the ManIID gene were integrated into *S. mutans* through natural transformation in the presence of 10 ng/ml of competence-stimulating peptide.

### Preparation of heat-killed *S. mutans*

*S. mutans* WT and Δ*pts* were grown in Todd-Hewitt broth with 2% yeast extract (THY) at 37°C for 6 h. After bacterial pellet was harvested by centrifugation, pellets were washed and resuspended in phosphate-buffered saline (PBS) and then incubated in a water bath at 70°C for 5 h. Complete inactivation was confirmed by spotting the bacterial suspension on agar plates. Bacterial pellets, wet weight-based, per colony-forming unit (CFU) was measured by spotting assay and fifty micrograms of each strain contained approximately 7.2 × 10^5^ CFU.

### Preparation of bone marrow-derived DCs

The animal study was reviewed and approved by the Seoul National University Institutional Animal Care and Use Committee (Approval Number: SNU-210403-1-2). Experiments were conducted in accordance with guidelines established by Seoul National University Institutional Animal Care and Use Committee. Bone marrow-derived DCs were prepared from 6 to 8-week-old mice. Briefly, mouse bone marrow cells were isolated by flushing femurs and tibiae with PBS. RBCs were removed by resuspending cell pellets with RBC lysis buffer. After lysis, bone marrow cells were differentiated into DCs by culturing with 20 ng/ml of GM-CSF in RPMI-1640 supplemented with 10% FBS and 1% penicillin-streptomycin for 8 days. Fresh culture medium containing 20 ng/ml of GM-CSF was added every 3 days.

### Examination of bacterial adherence and internalization

According to the previous studies, heat-killed bacteria preserved their surface structure and molecules, which are critical for bacterial adherence and internalization into host cells (Hament et al., 2004; Popović et al., 2019). Moreover, since use of heat-killed bacteria can eliminate variables that affect bacterial adherence and internalization into host cells, including bacterial growth, toxin production, and metabolic activity (Liang and Ji, 2006; Luqman and Ohlsen, 2025), heat-killed *S. mutans* strains were used in the current study for the examination of bacterial adherence and internalization into DCs. DCs (1 × 10^6^ cells/ml) were incubated in the presence or absence of mannan (3 mg/ml) for 10 min. DCs were washed with PBS 3 times to remove exogenous mannan. Then, heat-killed CFSE-labeled *S. mutans* WT and Δ*pts* (1, 10, 30, or 50 μg/ml) were incubated with DCs for 1 h at 4°C or 37°C for adherence and adherence/internalization. Adherence to and internalization of labeled bacteria into DCs were measured by CFSE intensity using flow cytometry (FACSVerse, BD Biosciences) as described previously (Kim et al., 2018; Ko et al., 2017). All flow cytometric data were analyzed using FlowJo software (TreeStar).

### Preparation of rManIID protein

To prepare a plasmid expressing ManIID, 854 bp of ManIID sequence (gene ID: EMP63015) was amplified by PCR using genomic DNA (gDNA) from *S. mutans* ATCC 25175. For PCR amplification, 200 ng of gDNA was amplified using a nPfu DNA polymerase (Enzynomics) with primers containing BamHI or AvaI site (Forward: 5’-GCGGGATCCATGGCTCAAAAGAA AATTTCAAA-3’; Reverse: 5’-GCGCYCGRGGTATTTCAAGCCTGGATCAAAGA-3’) under the following reaction condition: amplification by 30 cycles at 95°C for 30 s, 60°C for 30 s, and 72°C for 60 s. The PCR products were digested with BamHI and AvaI restriction enzymes (New England Biolabs) together with pET-21a plasmid containing a His-tag sequence (EMD bioscience) for 30 min at 37℃. The digested PCR products were cloned into the site between BamHI and AvaI in the multiple cloning sites of pET-21a plasmid using T4 DNA ligase (New England Biolabs) for 1 h at room temperature. The prepared ManIID plasmid was transformed into *E. coli* DH5α and selected using LB agar plates containing ampicillin. The plasmid was then isolated using the Plasmid Miniprep Kit (Qiagen). After the transformation of plasmid into *E. coli* BL21(DE3), bacteria were cultured in LB broth with 0.2 mM IPTG at 37°C for 16 h. Bacterial pellets were collected by the centrifugation and re-suspended in lysis buffer (50 mM monobasic sodium phosphate, 300 mM sodium chloride, and 10 mM imidazole, pH 8.0). After lysing the bacterial pellets using an ultra-sonicator (Sanyo), supernatants were collected and incubated with Ni-NTA agarose beads at 4°C for 2 h. The mixture was then packed into a column and washed with wash buffer (50 mM monobasic sodium phosphate, 300 mM sodium chloride, and 20 mM imidazole, pH 8.0). A series of fractions were further eluted with elution buffer (50 mM monobasic sodium phosphate, 300 mM sodium chloride, and 250 mM imidazole, pH 8.0). Based on the results from Coomassie blue staining followed by sodium dodecyl sulfate-polyacrylamide gel electrophoresis, the column fractions containing rManIID protein were pooled and used in further experiments.

### Analysis of DC maturation markers

DCs (1 × 10^6^ cells/ml) were treated with heat-killed *S. mutans* (1, 3, or 10 μg/ml) or rManIID (0.01, 0.1, or 1 μg/ml) for 48 h. After treatment, the cells were harvested and Fc receptors were blocked at 4℃ for 10 min to reduce non-specific binding. Cells were then stained with fluorochrome-conjugated antibodies specific to CD11c, CD80, CD86, MHC-I, or MHC-II for 20 min on ice and washed with PBS. CD11c-positive cells were regarded to be DCs and were used for analysis. Expression of DC maturation markers was analyzed by geometric mean fluorescence intensity (MFI) using flow cytometry, as described previously (Kim et al., 2023; Yang et al., 2009).

### Analysis of the endocytic capacity of DCs

The endocytic capacity of stimulated DCs was evaluated by measuring FITC-dextran uptake (Yang et al., 2009). Briefly, DCs (1 × 10^6^ cells/ml) were treated with heat-killed *S. mutans* (1, 3, or 10 μg/ml) for 48 h. After treatment, cells were harvested and washed with PBS. Cell pellets were resuspended in complete RPMI-1640 containing FITC-dextran (1 mg/ml) and incubated for 1 h at 4°C or 37°C to determine adherence and adherence/internalization of FITC-dextran. FITC-dextran uptake was measured by subtracting the flow cytometry-measured MFI value at 4°C from that at 37°C.

### Analysis of cytokine production

DCs (1 × 10^6^ cells/ml) were treated with heat-killed *S. mutans* (1, 3, or 10 μg/ml) or rManIID (0.01, 0.1, or 1 μg/ml) for 48 h. Then, the culture supernatant was harvested, and concentrations of IL-6 were measured with a commercial enzyme-linked immunosorbent assay (ELISA) kit (BioLegend) as described previously (Han et al., 2025; Kwabena Danso et al., 2024).

### Statistical analysis

All data are expressed as mean ± standard deviation (SD) of triplicate samples. Statistical significance was determined using Student’s *t*-test. An asterisk (*) indicates a significant difference, defined as *p* < 0.05.

## Results

### ManIID is involved in the adherence/internalization of *S. mutans* by DCs

**Fig. 1.**
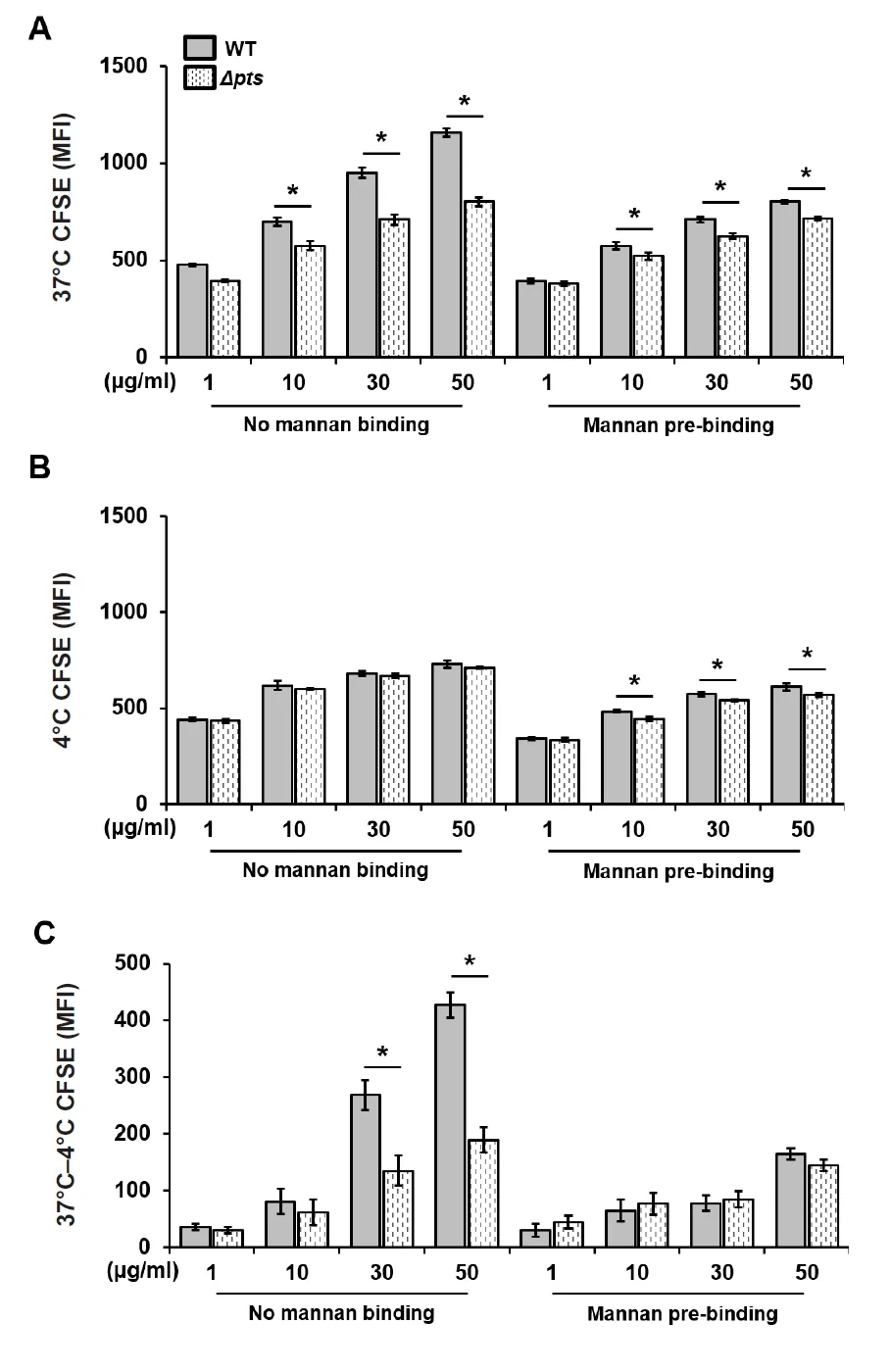
ManIID is involved in the adherence/internalization of *S. mutans* by DCs. DCs (1 × 10^6^ cells/ml) were incubated in the presence or absence of mannan (3 mg/ml) for 10 min. Then, DCs were incubated with heat-killed *S. mutans* WT or Δ*pts* (1, 10, 30, or 50 μg/ml) labeled with CFSE for 1 h at (A) 37°C and (B) 4°C. Adherence and internalization of the labeled bacteria to DCs were analyzed by measuring CFSE intensity using flow cytometry. (C) Specific internalization was calculated by subtracting the MFI at 4°C from that at 37°C (37°C–4°C). Results are presented as Mean ± SD of triplicate samples. Asterisks indicate statistical differences (*p* < 0.05) between WT and Δ*pts*.

DC maturation, a pivotal process needed for acquiring adaptive immunity, is initiated by bacterial capture and engulfment to process and present bacterial antigens to T cells (Xiao and Xia, 2023). Given that ManIID is one of the major sugar uptake systems in *Streptococcus* spp. (Jeckelmann and Erni, 2020), we hypothesized that recognition of *S. mutans* by DCs is accomplished by mannose receptors on DCs that recognize glycosylation patterns of proteins (van der Zande et al., 2021). To examine this, DCs were pre-bound with exogenous mannan for 10 min to block recognition by mannose receptors. Subsequently, DCs were incubated with CFSE-labeled heat-killed WT and Δ*pts* strains of *S. mutans* at 0, 1, 10, 30, or 50 μg/ml for 1 h at 4°C or 37°C to examine bacterial adherence and adherence/internalization, respectively. WT *S. mutans* displayed much higher adherence/internalization than the Δ*pts* strain in a dose-dependent manner in the non-mannan binding group (Fig. 1A). However, in the mannan pre-binding group, the adherence/internalization rate was substantially reduced, particularly for WT *S. mutans* (Fig. 1A). In contrast, at 4°C, WT and Δ*pts* *S. mutans* showed comparable adherence rates (Fig. 1B). When evaluating relative internalization by comparing the subtracted CFSE intensity (37°C–4°C), WT displayed a considerably higher internalization rate than Δ*pts* in a dose-dependent manner at 1 h after the treatment (Fig. 1C). Interestingly, mannan pre-binding resulted in similar internalization rates between the two strains (Fig. 1C). These results collectively demonstrate that the presence of ManIID plays a crucial role in the internalization of *S. mutans*, highlighting the significance of mannose receptors in this process.

### ManIID-deficient *S. mutans* induces less DC maturation than wild-type *S. mutans*

**Fig. 2.**
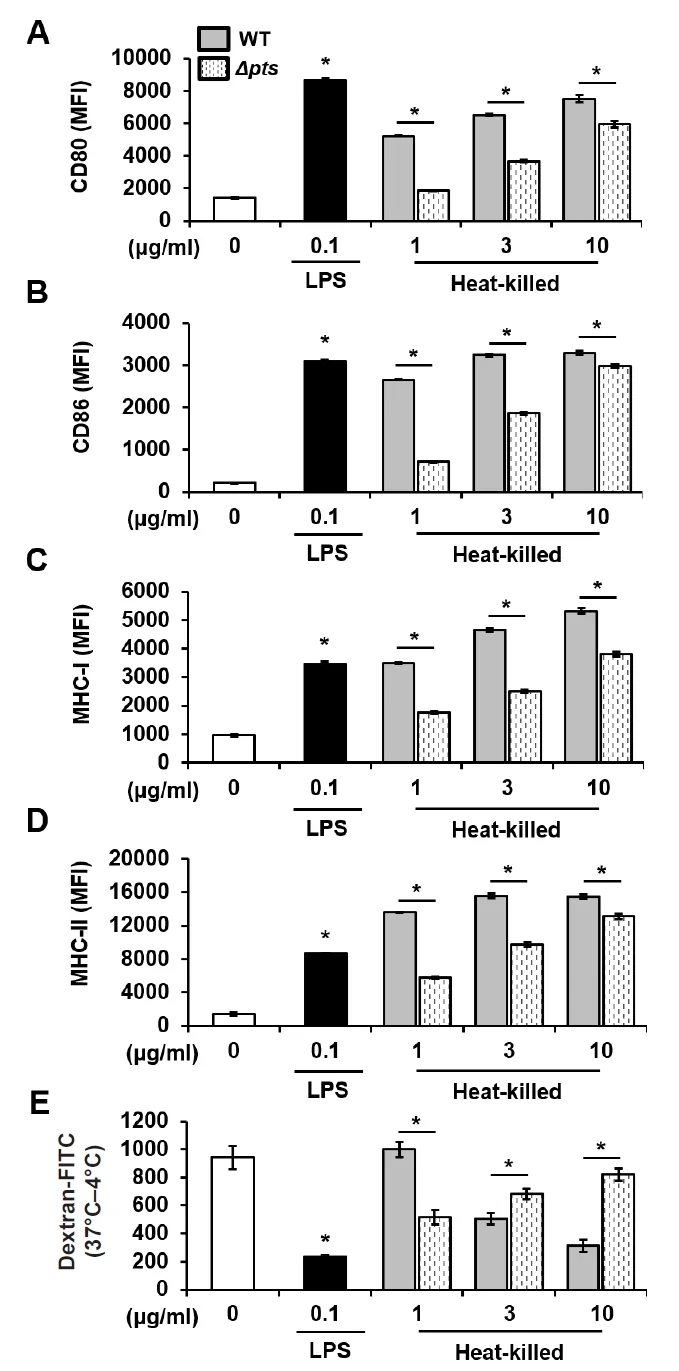
ManIID-deficient *S. mutans* induces less DC maturation than wild-type *S. mutans*. DCs (1 × 10^6^ cells/ml) were treated with WT or Δ*pts* heat-killed *S. mutans* at 1, 3, and 10 μg/ml for 48 h. LPS (0.1 μg/ml) was used as a positive control. After incubation, the expression levels of (A) CD80, (B) CD86, (C) MHC-I, and (D) MHC-II were analyzed by flow cytometry. (E) After incubation, DCs were collected and incubated with FITC-dextran (1 mg/ml) for 1 h at 37°C and 4°C. Uptake of FITC-dextran was measured by subtracting the 4°C MFI value from the 37°C MFI value. Bar graphs refer to the average MFI of phagocytic DCs. Results are presented as Mean ± SD of triplicate samples. Asterisks indicate statistical differences (*p* < 0.05) between WT and Δ*pts*, as well as between non-treatment and LPS. LPS, lipopolysaccharide.

DC maturation is accompanied by the upregulated expression of phenotypic maturation markers, such as CD80, CD86, MHC-I, and MHC-II (Kim et al., 2007; Lapteva et al., 2007). Thus, to evaluate the contribution of ManIID to DC maturation, DCs were treated with heat-killed WT or ManIID-deficient strain (Δ*pts*) of *S. mutans* at 0, 1, 3, or 10 μg/ml and expression of the maturation markers was examined. As shown in Fig. 2A–2C, DCs treated with Δ*pts* expressed relatively lower levels of CD80, CD86, MHC-I, and MHC-II on their surfaces than those treated with the wild-type strain. Moreover, the decrease in maturation marker expression in response to Δ*pts* strain treatment was greatest at 1 μg/ml. To investigate the ManIID-induced maturation of DCs in more detail, a FITC-dextran uptake assay was performed, as endocytosis of DCs declines progressively with maturation (Kim et al., 2008; Reis e Sousa et al., 1999). Consistent with the marker expression results, DCs treated with WT dose-dependently lost their endocytosis ability while endocytosis of the △*pts* strain was comparable to that of untreated DCs (Fig. 2E). Collectively, these results indicate that ManIID contributes substantially to DC maturation in the presence of *S. mutans*.

### ManIID promotes the maturation marker expression by DCs

**Fig. 3.**
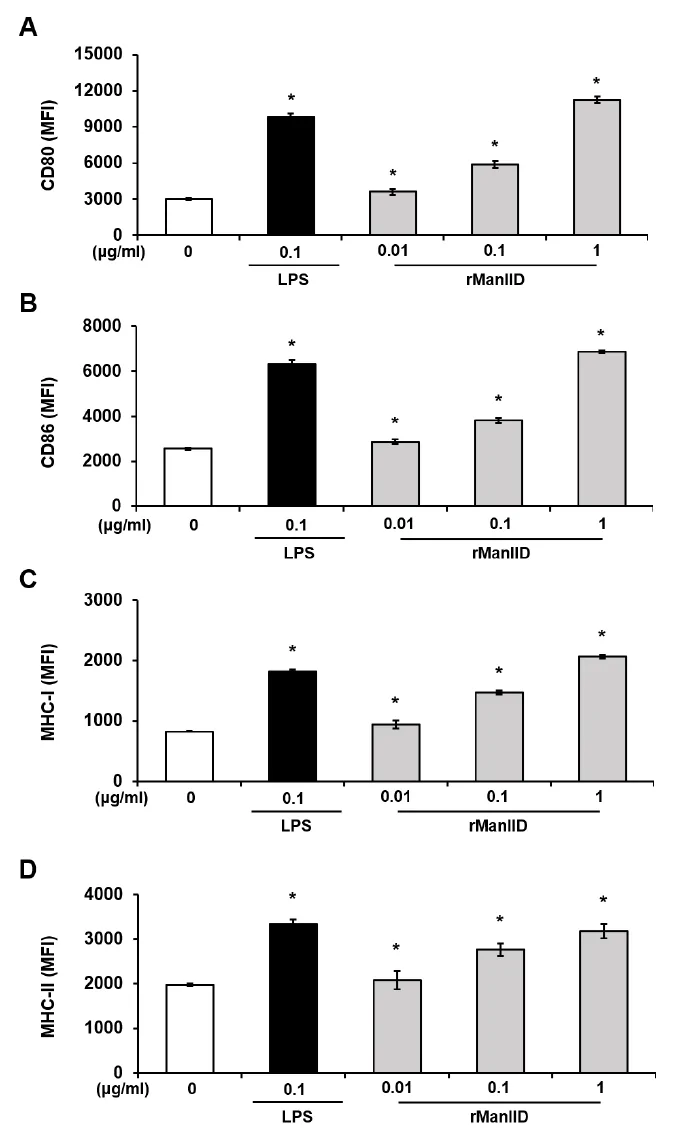
ManIID promotes the maturation marker expression by DCs. DCs (1 × 10^6^ cells/ml) were treated with rManIID at 0.01, 0.1, and 1 μg/ml for 48 h. LPS (0.1 μg/ml) was used as a positive control. After incubation, the expression levels of (A) CD80, (B) CD86, (C) MHC-I, and (D) MHC-II were analyzed by flow cytometry. Results are presented as Mean ± SD of triplicate samples. Asterisks indicate statistical differences (*p* < 0.05) between non-treatment and LPS or rManIID. LPS, lipopolysaccharide; rManIID, recombinant ManIID protein.

To further confirm the effects of ManIID on DC maturation, DCs were treated with rManIID at 0, 0.01, 0.1, or 1 μg/ml for 48 h. As shown in Fig. 3A–3D, rManIID dose-dependently increased the expression of maturation markers including CD80, CD86, MHC-I, and MHC-II. Also, treatment of DCs with ManIID at 1 μg/ml resulted in greater maturation marker expression (CD80, CD86, and MHC-I) than that induced by LPS, highlighting the profound immunostimulatory activity of ManIID (Fig. 3A–3C). Taken together, rManIID markedly promotes DC maturation suggesting that ManIID may be the principal component of *S. mutans* that activates DCs.

### ManIID stimulates robust IL-6 production by DCs

**Fig. 4.**
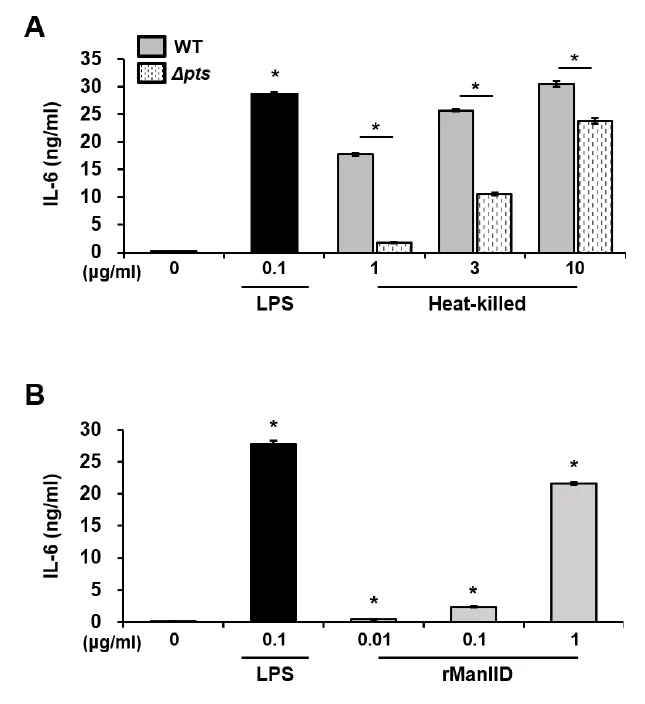
ManIID stimulates robust IL-6 production by DCs. (A) DCs (1 × 10^6^ cells/ml) were treated with WT or Δ*pts* heat-killed *S. mutans* at 1, 3, and 10 μg/ml for 48 h. (B) DCs (1 × 10^6^ cells/ml) were treated with rManIID at 0.01, 0.1, and 1 μg/ml for 48 h. LPS (0.1 μg/ml) was used as a positive control. After incubation, culture supernatant was collected, and the amount of IL-6 was measured by ELISA. Results are presented as Mean ± SD of triplicate samples. Asterisks indicate statistical differences (*p* < 0.05) between WT and Δ*pts*, as well as between non-treatment and LPS or rManIID. LPS, lipopolysaccharide; rManIID, recombinant ManIID protein.

Once activated, DCs produce various cytokines, such as TNF-α, IL-6, and IL-12 for triggering the adaptive immune response (Yin et al., 2021). To evaluate the effects of ManIID on DC maturation and activation, DCs were treated with heat-killed WT and Δ*pts* *S. mutans* at 0, 1, 3, or 10 μg/ml for 48 h, and culture supernatant was collected to measure IL-6 production by ELISA. In addition, DCs were treated with 0.01, 0.1, and 1 μg/ml rManIID, and IL-6 production was measured. In accordance with maturation marker expression patterns, WT elicited greater IL-6 production than Δ*pts* in a dose-dependent manner (Fig. 4A). The difference in IL-6 production between the two strains was most pronounced at 1 μg/ml (Fig. 4A). Furthermore, rManIID also dose-dependently promoted IL-6 production by DCs (Fig. 4B). These results indicate that ManIID is a key immunostimulatory component that elicits robust inflammatory cytokine production by DCs.

## Discussion

*S. mutans* is the major causative pathogen of various human dental and systemic diseases, including dental caries, pulpitis following deep caries and infective endocarditis (Hahn and Liewehr, 2007; Matsumoto-Nakano, 2018; Nobbs, 2017). Although DCs are important for progression of *S. mutans*-caused infectious diseases and related immune responses (Butcher et al., 2011; Hahn et al., 2005), the component of *S. mutans* responsible for the DC responses has yet to be identified. In the present study, internalization of *S. mutans* into DCs was mediated by ManIID expressed on the surface of *S. mutans*. Additionally, mannan pre-binding of DCs led to comparable internalization between the WT and Δ*pts* strains, suggesting that the mannose receptors of DCs are important sensors that mediate the interaction between DCs and *S. mutans*. Furthermore, deletion of ManIID considerably attenuated the expression of phenotypic maturation markers and cytokine production by DCs, indicating that ManIID is a key immuno-stimulating component of *S. mutans* that triggers DC activation. Moreover, stimulation with rManIID also induced the expression of DC maturation markers, further supporting its immunostimulatory properties.

In the current study, rManIID directly stimulated DCs leading to their maturation and activation. According to previous studies, other membrane proteins of *S. mutans*, namely wall-associated protein A and saliva-binding regions of adhesion AgI/II, can also induce the expression of phenotypic maturation markers, including CD80, CD86, and MHC-II, and proinflammatory cytokine, such as IL-6 (Li and Wang, 2014; Xu et al., 2011). However, since the capacity of these other membrane proteins to activate DCs is relatively weaker than that of ManIID, ManIID is likely the major etiological component of *S. mutans* responsible for DC activation. The relatively higher DC activation capacity of ManIID might be due to its structure. In more detail, ManIID possesses highly variable periplasmic loops that transport sugars (Jeckelmann and Erni, 2020) which can potentially interact with surface receptors of DCs to elicit responses. It has been reported that ManIID can bind and form a complex with other proteins from bacteriocin-producing bacteria, such as immunity protein PedB, which protects them from their own bacteriocin (Zhou et al., 2016). To clarify the exact mechanisms responsible for ManIID-induced DC activation, the aforementioned possibility should be verified through further studies.

The maturation and activation of DCs by ManIID potentially arise from differences in internalization rates mediated by mannose receptor recognition. Our results indicated that mannose receptors are crucial in mediating the adherence of *S. mutans* to DCs, as evidenced by the pre-binding assay using mannan (Fig. 1). Due to their ability to bind and internalize various endogenous and pathogen-associated molecules, the role of mannose receptors such as CD206 has been extensively evaluated (van der Zande et al., 2021). For example, soluble mannose receptors potentiate the inflammatory responses of macrophages, as reflected by elevated proinflammatory cytokine secretion and glycolysis (Embgenbroich et al., 2021). Furthermore, pathogens including *Candida albicans*, *Mycobacterium tuberculosis*, and *K. pneumoniae* are efficiently removed by mannose receptor of exposed sugar moieties on the surface of these microbes (van der Zande et al., 2021). Therefore, mannose receptors can be utilized by DCs to recognize sugar moieties abundantly expressed on the surface of *S. mutans*.

On the other hand, the altered biosynthesis and structure of extracellular polysaccharide (EPS) matrix in *S. mutans* Δ*pts* strain might affect its bacterial adherence and internalization into DCs. Unlike the other streptococci, *S. mutans* possesses EPS matrix instead of a polysaccharide capsule as its functional analogue which is involved in its adherence and internalization (Alves et al., 2016; Lemos et al., 2019). Moreover, the previous studies suggested a possibility that ManIID can be supposedly involved in biosynthesis and structure composition of *S. mutans* EPS matrix. In fact, mutations in *S. mutans* Man-PTS subunits reduced the uptake of sugar precursors for EPS biosynthesis and expression of glucosyltransferases responsible for EPS synthesis thereby impairing EPS matrix quantity and structure (Abranches et al., 2006; Moye et al., 2014). Thus, to clarify the aforementioned possibility, the roles of ManIID in the biosynthesis and structure of EPS matrix of *S. mutans* should be verified through further studies.

In the current study, ManIID was demonstrated to play a crucial role in bacterial internalization into DCs and act as a key immuno-stimulating agent responsible for DC activation. According to previous studies, oral streptococci commonly possess Man-PTS as a sugar transporter on their surface. In fact, Man-PTS has been detected in most oral pathogenic streptococci including *S. mutans*, *Streptococcus gordonii*, *Streptococcus sanguinis*, *Streptococcus salvivarius*, and *Streptococcus pyogenes*, all of which are causative bacteria for dental caries, periodontitis, pulpitis and gingivitis (Marple et al., 2025; Vadeboncoeur and Pelletier, 1997). Moreover, when the amino acid sequence of *S. mutans* ManIID was compared with those of other oral streptococci, including *S. gordonii*, *S. sobrinus*, *S. sanguinis*, *Streptococcus oralis*, and *Streptococcus mitis*, by a sequence alignment analysis using CLC Sequence Viewer, all of these oral streptococci share similar ManIID amino acid sequences with that of *S. mutans* (data not shown). In addition, a recent study demonstrated that various glycolysis pathways, including pentose, glucuronate, fructose, and mannose pathways, were enriched in the subgingival microbiome during periodontitis development, suggesting that ManIID contributes to the severity of periodontitis (Lu et al., 2022). Thus, ManIID appears to be a major etiological component of oral streptococci that plays an important role in the initiation and progression of diverse dental diseases caused by streptococcal infection.

*S. mutans* is a key causative pathogen of dental caries (Lemos et al., 2019; Matsumoto-Nakano, 2018), and pulpal infection by this bacterium following deep caries has been linked to the initiation and progression of pulpitis by increasing dental pulp inﬂammation (Quispe-Salcedo and Ohshima, 2021). During the initial stage of bacterial infection in pulpal tissue as dental caries develop, DCs play an important role in eliminating invading bacteria through antigen presentation to lymphocytes and cytokines production, bridging the gap between host innate and adaptive immunity (Harmon et al., 2009; Horst et al., 2011). Despite the important roles of DCs in *S. mutans* infection, little is known about the interaction between *S. mutans* and DCs (Butcher et al., 2011; Hahn et al., 2005). Here, we found that ManIID plays a crucial role in the internalization of *S. mutans* into DCs and acts as a key immune-stimulating agent responsible for DC responses during *S. mutans* infection. Given its immunological properties, ManIID warrants further investigation as a viable therapeutic target in future studies aimed at combating *S. mutans*-related pathogenicity and associated diseases, especially pulpitis secondary to deep caries.
